# Myocardial ^123^I-MIBG Uptake and Cardiovascular Autonomic Function in Parkinson's Disease

**DOI:** 10.1155/2015/805351

**Published:** 2015-11-16

**Authors:** Akira Katagiri, Masato Asahina, Nobuyuki Araki, Anupama Poudel, Yoshikatsu Fujinuma, Yoshitaka Yamanaka, Satoshi Kuwabara

**Affiliations:** ^1^Department of Neurology, Chiba University School of Medicine, Chiba 260-8670, Japan; ^2^Department of General Medical Science, Chiba University School of Medicine, Chiba 260-8670, Japan

## Abstract

*Introduction*. Patients with Parkinson's disease (PD) showed reduced myocardial ^123^I-MIBG uptake, which may affect autonomic regulation. We investigated correlation between MIBC accumulation and cardiovascular autonomic function in PD.* Methods*. We performed myocardial MIBG scintigraphy, heart rate variability (HRV) analysis, and the head-up tilt test (HUT) in 50 PD patients (66.4 ± 7.8 years; duration 5.5 ± 5.9 years). Autonomic function tests were also performed in 50 healthy controls (66.5 ± 8.9 years). As HRV parameters, a high-frequency power (HF, 0.15–0.4 Hz), a low-frequency power (LF, 0.04–0.15 Hz), and LF/HF ratio were used.* Results*. Our PD patients had a significant reduction in LF and HF compared with the controls (*P* = 0.005 and *P* = 0.01). In HUT, systolic and diastolic blood pressure falls in the PD group were significantly greater than those in the controls (*P* = 0.02 and *P* = 0.02). The washout rate of MIBG was negatively correlated with blood pressure changes during HUT.* Conclusion*. Our PD patients showed reduced HRV, blood pressure dysregulation, and reduced MIBG accumulation, which was correlated with blood pressure dysregulation. Orthostatic hypotension in PD may be mainly caused by sympathetic postganglionic degeneration.

## 1. Introduction

In patients with Parkinson's disease (PD), nonmotor symptoms such as hyposmia, mood disorders, sleep disorders, and autonomic abnormalities often precede motor symptoms, and *α*-synuclein deposition in the olfactory bulb, enteric plexus, or dorsal motor nucleus of the vagus nerves is considered to proceed to nigral degeneration [1]. In addition, ^123^I-metaiodobenzylguanidine (MIBG) scintigraphy reveals that cardiac postganglionic sympathetic neurons are involved in the early or premotor stage of PD [2]. Lesions in the dorsal motor nucleus, which mediate cardiac parasympathetic activity, and postganglionic sympathetic neurons could affect heart rate or blood pressure regulation. On the other hand, blood pressure regulation is clinically evaluated by the head-up tilt test, and spectral analysis of heart rate variability (HRV) is a noninvasive method for studying heart rate regulation in relation to both sympathetic and parasympathetic inputs. The power spectral density of R-R intervals is generally categorized into high-frequency (HF) and low-frequency (LF) components. The HF (baroreflex) component has been attributed to parasympathetic vagal modulation (respiratory sinus arithmetic component), whereas the LF component appears to be produced by baroreflex feedback, which is mainly modulated by sympathetic activities, although vagal parasympathetic tone may partially affect the LF component [3–5]. Spectral HRV analysis may detect cardiac autonomic dysfunction due to degeneration of vagal neurons or cardiac postsynaptic sympathetic neurons in PD. We performed myocardial MIBG scintigraphy, head-up tilt test, and spectral HRV analysis in patients with PD to reveal correlation between cardiac sympathetic denervation and heart rate or blood pressure regulation.

## 2. Materials and Methods

### 2.1. Subjects

We studied 50 patients with PD (mean age, 66.4 ± 7.8 years; 23 men and 27 women). All patients with PD met the United Kingdom Brain Bank criteria for PD [6]. The exclusion criteria included cardiac disorders, kidney or liver diseases, endocrine dysfunction, malnutrition, peripheral neuropathy, diabetes, severe hypertension, or dementia. The mean disease duration was 5.5 ± 5.9 years (range, 0.5–21 years). The mean Hoehn and Yahr score was 2.3 ± 0.9 (range, 1–4). Thirty patients with PD were taking levodopa plus a decarboxylase inhibitor (L-dopa/DCI); 11 were taking L-dopa/DCI alone, and 19 received a combination of L-dopa/DCI and dopamine agonist. Three patients were treated with a dopamine agonist alone. Seventeen patients are not treated with any antiparkinsonian drugs. The mean L-dopa equivalent daily dose [7] was 333.6 ± 368.9 mg/day. No patient was receiving anticholinergic drugs. Fifty healthy control subjects (mean age, 66.5 ± 8.9 years, 28 men and 22 women) were also examined. None were taking any medications that could affect autonomic function or had any neurologic disorders or clinically significant illnesses, including cardiac disorders, kidney or liver diseases, endocrine dysfunction, malnutrition, peripheral neuropathy, diabetes, severe hypertension, or dementia. Informed consent was obtained from all participants. The ethics committee of Chiba University School of Medicine reviewed and authorized the protocol.

### 2.2. MIBG Myocardial Scintigraphy and Autonomic Function Tests

MIBG myocardial scintigraphy was performed in all patients with PD. Planar scintigraphic images in the anterior view were captured by a single-head gamma camera (GCA-7200A/UI, Toshiba, Japan) 15 min (early) and 3 h (delayed) after intravenous injection of MIBG (111 MBq). To measure MIBG uptake, heart (left ventricle) and mediastinal regions of interest were drawn manually. The heart to mediastinum ratio (H/M) for both early and delayed images and the myocardial washout rate for delayed images were calculated. The normal ranges of MIBG parameters are >2.09 for H/M in delay phase and <33% washout rate in our institution (data not shown). For all participants, scintigraphy was performed within 1 month (before or after) of HRV analyses.

The head-up tilt test and HRV analysis were performed in patients with PD and healthy control subjects in a quiet room at an ambient temperature of 22–26°C. Each subject was asked to relax, stay awake, and remain in a supine position for at least 15 min before each test. During the head-up tilt test, blood pressure and heart rate were measured using a sphygmomanometer at 1-min intervals. After 5 min of baseline measurement, each subject was passively tilted on an electrically driven tilt table to 70° for 10 min. Orthostatic hypotension was defined as a decrease in systolic blood pressure of at least 20 mmHg or diastolic blood pressure of at least 10 mmHg within 3 min of tilting [8]. For HRV analysis, the electrocardiogram was recorded with the subject in a supine position, and at least 300 consecutive R-R intervals were measured during normal breathing at a sampling rate of 1000 Hz. As an index of time-domain analysis, the coefficient of variation of the R-R intervals (CV_R-R_) was calculated as the standard deviation divided by the mean of 100 R-R intervals. The average of three CV_R-R_ values was used. An abnormal CV_R-R_ value was judged according to age-matched data from our laboratory (not shown). The power spectrum analysis of 300 R-R intervals was computed using fast Fourier transformation. The power spectrum was quantified into frequency-domain measurements; high-frequency oscillations (HF, 0.15–0.4 Hz) and low-frequency oscillations (LF, 0.04–0.14 Hz) were estimated from the spectra. In addition, the ratio between LF and HF (LF/HF) was calculated [5].

### 2.3. Statistical Methods

The Mann-Whitney *U* test was used to analyze mean differences between the PD and control groups. Spearman correlation coefficients were used to examine the relationships between disease duration, modified Hoehn and Yahr score, or L-dopa equivalent daily dose and the results of MIBG myocardial scintigraphy and autonomic function tests. Differences were considered statistically significant when *P* values were <0.05.

## 3. Results

**Figure 1:**
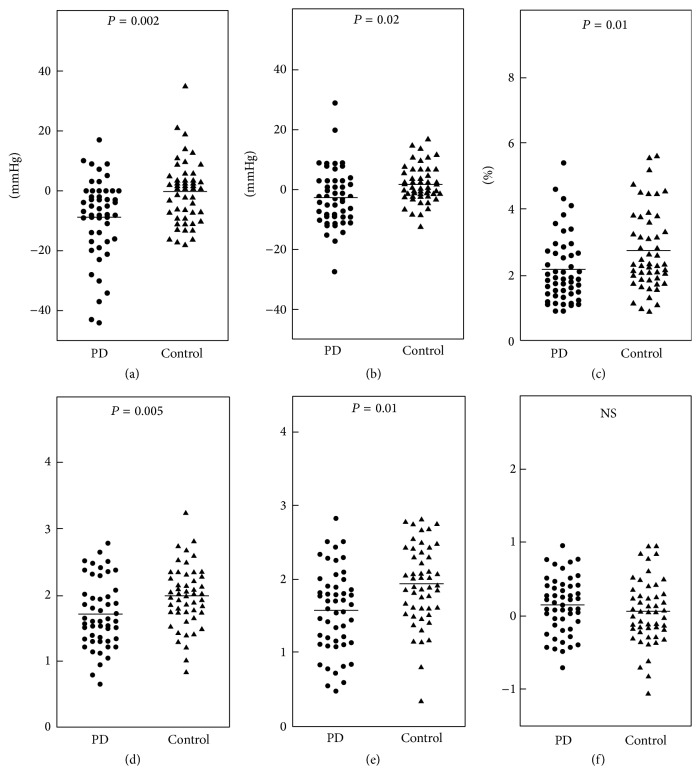
Systolic (a) and diastolic (b) blood pressure changes during the head-up tilt test, CV_R-R_ (c), low-frequency (LF) component (d), high-frequency (HF) component (e), and LF/HF ratio (f) in patients with Parkinson's disease (PD) and healthy control subjects. The values of LF, HF, and the LF/HF ratio are presented as logarithmic transformations. Bars indicate means.

In MIBG myocardial scintigraphy, 39 patients with PD (78%) showed a decrease in the H/M ratio on delay phase images, and 42 patients (84%) exhibited an increased washout rate. The mean H/M ratios in the early and delayed phases were 2.05 ± 0.68 and 1.84 ± 0.88, respectively, and the mean washout rate was 56.8 ± 18.7% in patients with PD. In the head-up tilt test, 11 patients with PD (22%) had orthostatic hypotension, whereas no control subjects had orthostatic hypotension. There were no significant differences in the means of systolic and diastolic blood pressure in the supine position between the PD (121.8 ± 18.9/66.8 ± 10.0 mmHg) and control (123.8 ± 19.1/72.4 ± 12.6 mmHg) groups.  Figure 1 shows the results of the head-up tilt test and heart rate variability in the PD and control groups. A mean fall in systolic blood pressure during the head-up tilt test in the PD group (−8.8 ± 13.3 mmHg) was significantly greater than those in the control group (0 ± 7.4 mmHg, *P* = 0.002), and a mean fall in diastolic blood pressure in the PD group (−2.5 ± 9.6 mmHg) was also significantly greater than those in the control group (1.9 ± 5.6 mmHg, *P* = 0.02). A mean CV_R-R_ value in the PD group (2.16 ± 1.03%) was significantly lower than that in the control group (2.33 ± 0.76%, *P* = 0.01). A mean LF component in the PD group (99.5 ± 127.5 ms^2^) was significantly lower than that in the control group (121.1 ± 115.7 ms^2^, *P* = 0.005). A mean HF component in the PD group (79.3 ± 115.6 ms^2^) was also significantly lower than that in the control group (100.5 ± 123.0 ms^2^, *P* = 0.01). No significant difference was observed in the LF/HF ratio between the PD (2.0 ± 1.8) and control groups (2.5 ± 2.4).

Table 1 presents the Spearman correlation coefficients between the disease parameters and results of MIBG myocardial scintigraphy and autonomic function tests. No significant correlations were revealed.  Table 2 presents the Spearman correlation coefficients between the results of MIBG myocardial scintigraphy and autonomic function tests. The washout rate was negatively correlated with systolic (*P* = 0.02) and diastolic (*P* = 0.04) blood pressure changes during the head-up tilt test but not with any parameter derived from HRV analysis.

## 4. Discussion

Decreased accumulation of MIBG, which indicates sympathetic denervation of the heart, has been reported even in the early stages of PD [2]. In our patients with PD, cardiac accumulation of MIBG was reduced, and LF power derived from HRV analysis was significantly reduced. LF mainly reflects baroreflex feedback, which is driven by sympathetic activation. Therefore, LF power in our patients with PD indicates reduced cardiac sympathetic activity and may reflect cardiac postganglionic sympathetic denervation, which is demonstrated by myocardial MIBG scintigraphy. However, we found no significant correlation between the LF component and myocardial MIBG accumulation as well as results in a previous study [9]. Myocardial MIBG uptake has also been reported to be reduced in diabetic patients with normal HRV [10]. As possible explanations, the detection sensitivity of HRV analysis may be lower than that of myocardial MIBG scintigraphy, or functional deficits in sympathetic heart rate regulation may not be simply proportional to postganglionic cardiac denervation.

Postmortem studies of the premotor phase of PD found that *α*-synuclein pathology appeared in the dorsal motor nucleus of the vagus nerve [11] as well as the anterior olfactory structures [11] and the enteric nervous system [1], suggesting that parasympathetic vagal afferent is also impaired in the early or premotor phase of PD. The degeneration of the dorsal motor neurons may reduce the HF component, which is considered to be attributed to parasympathetic vagal modulation. In fact, our patients with PD showed mildly but significantly reduced HF power. Previous studies have presented conflicting results on dysfunctional parasympathetic cardioregulation in early PD, with two independent studies showing a significantly decreased HF component in PD [12, 13] and another showing no significant HF changes [14]. Compensatory recovery of vagal control has been reported after unilateral vagotomy [15]. The compensatory mechanism relieves vagal dysfunction due to the medullary neuropathology; therefore, severe HF reduction may not occur, particularly in the early phase of PD.

In our study, patients with PD showed a significant negative correlation between the myocardial washout rate of MIBG and blood pressure changes during the head-up tilt test. One previous study showed a significantly greater decrease in myocardial MIBG uptake in patients with PD with orthostatic hypotension during the head-up tilt test [16]; however, another study did not [9]. Blood pressure regulation in response to orthostatic stress is mainly mediated by sympathetic nerve activity, which induces peripheral vasoconstriction, particularly in the lower legs. Therefore, cardiac sympathetic denervation may not necessarily cause orthostatic hypotension. The reduced myocardial MIBG uptake in our patients may have reflected generalized sympathetic denervation in PD.

In our present study, 33 out of 50 patients with PD were treated with antiparkinsonian agents, including L-dopa (a dopamine precursor). Since dopamine is structurally related to norepinephrine, competitive MIBG uptake inhibition (depletion of granules) has been suspected [17]. In addition, antiparkinsonian medication may cause autonomic dysfunction [18]. Although the mean L-dopa equivalent daily dose in our patients with PD was not high, the limitations of our present study include medication.

## 5. Conclusion

Our patients with PD showed reduced LF and HF, blood pressure dysregulation, and reduced myocardial MIBG accumulation, which was correlated with severity of orthostatic hypotension. Orthostatic hypotension in PD may be mainly caused by sympathetic postganglionic degeneration.

## Figures and Tables

**Table 1:** Correlation between disease parameters and MIBG scintigraphy or autonomic function tests.

	Disease duration (*P* value)	H&Y (*P* value)	LEDD (*P* value)
MIBG scintigraphy			
Early H/M ratio	−0.10 (0.49)	−0.14 (0.32)	0.07 (0.63)
Late H/M ratio	−0.06 (0.66)	−0.15 (0.31)	0.11 (0.45)
Washout rate	0.04 (0.81)	0.19 (0.20)	−0.03 (0.85)
Autonomic function tests			
HRV			
LF component	−0.11 (0.44)	−0.08 (0.60)	−0.08 (0.57)
HF component	−0.26 (0.07)	−0.20 (0.17)	−0.17 (0.25)
LF/HF ratio	0.16 (0.28)	0.09 (0.55)	0.11 (0.43)
CV_R-R_	−0.11 (0.49)	−0.09 (0.32)	−0.06 (0.68)
HUT test			
SBP change	0.03 (0.84)	−0.04 (0.78)	0.00 (0.98)
DBP change	0.18 (0.22)	0.10 (0.50)	0.08 (0.58)
HR change	0.09 (0.52)	−0.12 (0.43)	0.12 (0.39)

Data are represented as Spearman correlation coefficients. H&Y, Hoehn and Yahr stage; LEDD, L-dopa equivalent daily dose; MIBG, ^123^I-metaiodobenzylguanidine myocardial scintigraphy; H/M ratio, heart to mediastinum ratio; HRV, heart rate variability; LF, low-frequency; HF, high-frequency; CV_R-R_, coefficient of variation of R-R intervals; HUT, head-up tilt; SBP, systolic blood pressure; DBP, diastolic blood pressure; HR, heart rate.

**Table 2:** Correlation between MIBG scintigraphy and autonomic function tests.

	Early H/M ratio (*P* value)	Delayed H/M ratio (*P* value)	Washout rate (*P* value)
HRV			
LF component	0.02 (0.89)	−0.02 (0.90)	0.00 (0.99)
HF component	0.18 (0.21)	0.13 (0.35)	−0.20 (0.17)
LF/HF ratio	−0.16 (0.28)	−0.16 (0.27)	0.26 (0.07)
CV_R-R_	0.21 (0.15)	0.19 (0.19)	−0.27 (0.06)
HUT test			
SBP change	0.17 (0.23)	0.24 (0.09)	**−0.34 (0.02)**
DBP change	0.07 (0.63)	0.14 (0.32)	**−0.29 (0.04)**
HR change	0.20 (0.16)	0.15 (0.30)	0.12 (0.43)

Data are represented as Spearman correlation coefficients. The bold letter indicates significant (*P* < 0.05) correlation. MIBG, ^123^I-metaiodobenzylguanidine myocardial scintigraphy; H/M ratio, heart to mediastinum ratio; HRV, heart rate variability; LF, low-frequency; HF, high-frequency; CV_R-R_, coefficient of variation of R-R intervals; HUT, head-up tilt; SBP, systolic blood pressure; DBP, diastolic blood pressure; HR, heart rate.
